# Malarial Poisoning

**Published:** 1883-12

**Authors:** Wm. Johnstone Fyffe

**Affiliations:** Deputy Surgeon-General; President Bristol Medico-Chirurgical Society, October, 1883


THE BRISTOL

### Medico-Chirurgical Journal.

DECEMBER, 1883.

### MALARIAL POISONING.

### Inaugural Address

BY

WM. JOHNSTONE FYFFE, M.D.,

*Deputy Surgeon-General;*

*President Bristol Medico-Chirurgical Society, October, 1883.*

GENTLEMEN,

I beg to thank you very cordially for the honor you have done me in electing me to the office of President of the Bristol Medico-Chirurgical Society.

It has been the custom for the new President of each year to deliver an inaugural address upon some subject of medical or surgical science. To me it has been a matter of some difficulty to select a topic of sufficient interest without going over ground which has been fully and ably discussed by others many times over. The mine seems at first sight to be almost exhausted, and yet how vast is the undiscovered land to those who are seekers after Nature's truths. We live in days when busy brains are searching with all their powers for the ultimate causes of specific diseases, when the investigation for living organisms in the blood and animal tissues has become the absorbing object of modern pathological enquiry.

Professor Tyndall exclaims with characteristic enthusiasm: "The physician and the sanitarian have no longer to fight against phantoms—their enemy is revealed. It is not noxious gases, but organized germs—contagia are living things. Men and women have died that bacteria and bacilli might live!"

This is strong language!—ought we not to receive it with some caution, and, at all events, wait for further experiment and examination? Take for example the now famous discovery of the tubercle bacillus by Koch. No one doubts its existence, or that it is a particulate and demonstrable fact. But are we certain that the deductions drawn from its discovery are true? Is it yet a settled question whether it is the cause or the consequence of phthisis? Does not the subject require still more elucidation before we can accept it as an incontrovertible basis for a theory of the causation of consumption?

All honor however to the worker! He has certainly added another solid stone to the arch which may yet be filled in and completed, he has erected another sign post which may yet guide us to the end. Thus it is "that in the discovery of truth of every kind, in the development of man's powers and privileges, each generation has its assigned part, and it is for us to endeavour to perform our portion of this perpetual task to our species."

To do this in spite of failure, to add spark by spark to the light until darkness and doubt pass away, is, and ever will be, the highest aim of the students of medical science.

May I trespass on your patience and ask you to follow me for a little, while I direct your attention to a view of the present state of our knowledge with regard to the nature of the malarial poison, and to some of its pathological effects upon the human organism. The subject is still in many respects clouded in mystery, but there are a certain number of tolerably well authenticated facts belonging to it, which we may appropriate, and by gathering them together we may be able to obtain a view of the question which I hope may not be without some interest to you.

It would be impossible, I need not say, in the brief space at my disposal to enter into any exhaustive discussion of this question. It would require a whole course of lectures to unfold and unravel the different aspects which are presented to our view in studying the subject of malarial poisoning. Its geographical distribution; its strange clinging to certain regions and districts; the perplexity which meets us when we try to fix its origin and charge it upon the assemblage together of certain physical facts in certain localities; that no race, nationality, or colour, enjoy any immunity from its influence; that all periods of life are liable to it, even down to the infant *in utero*; that some of the fairest and loveliest places on the earth are rendered well nigh uninhabitable by it—are all notorious and well ascertained facts. I need not enlarge upon them; I would rather select from among the vast amount of material which has been collected, one or two prominent and salient features which seem to show that in the study of malaria we are dealing with a poison germ, an active *materies morbi.*

Some observers have gone so far as to state that there is no such thing as malaria in existence. They regard intermittent and remittent fever, cholera and insolation, as manifestations of one great type of fever; they declare that telluric emanations have nothing to do with these fevers, and that the whole of their history and geographical distribution, their etiology, and destructive effects, are explicable on the hypothesis of alternations of temperature suddenly acting upon the human body.

What is malaria? The question is not yet answered, but I for one do not doubt that it will be. An enormous impetus has been given of late years to the investigation of the ultimate factors of acute febrile diseases. Who can set limits to the work yet to be done by such manipulators as Pasteur, Koch and others?

Efforts have not been wanting thus to search for the special poison germ of malaria. Since the days of Varro —a century before Christ—down to the present time, investigations have been made to solve the riddle: low forms of vegetable life, the algæ of the Pontine marshes, emanations from the soil in the form of gases forced out by the rise and fall of the subsoil water—these in their turn have been considered the factors in causing the phenomena of malarial poisoning.

A few years ago two distinguished workers, Klebs and Tommasi-Crudeli, conducted a series of investigations into the question. They found bacilli in the air and water of the Roman marshes, which they submitted to a process of cultivation. The results are thus described by Sir Joseph Fayrer:—

Rabbits inoculated with washings from the soil, or with fluids in which the bacillus has been cultivated, suffered from intermittent fever.

Filtered liquids caused only slight symptoms.

All the animals with fever had great enlargement of the spleen.

Many of the spleens exhibited pigmentation.

The bacilli were found in the spleens and marrow of the animals as well as in the soil.

The spores of the bacillus were, according to Crudeli, constantly found in the blood of the infected rabbits.

Venous blood from ague patients gave intermittent fever to dogs when introduced into the veins. Crudeli considers that the rigors of ague are produced by the irritation of the bacilli upon the vaso-motor centres.

It would seem at first sight, after these experiments and those of others in France, Italy and Algeria, that the mystery had been solved at last, and the crowning triumph seemed to have been reached when Dr. Aitken, of Rome, exhibited specimens of malarial bacillus at the meeting of the British Medical Association at Cambridge. But, alas! for the fate of this theory, other workers appeared, both from the United States and India. A medical officer of the United States army, Dr. Sternberg, found in the soil, water and air of New Orleans (the head-quarters of malaria) algæ and bacteriæ in various forms, some resembling those of Klebs and Crudeli, and they found also that they were capable under cultivation of acquiring virulent properties when injected into the blood of some of the lower animals, but that they do *not* produce the peculiar paroxysmal phenomena of intermittent fever. He found that the experiments on rabbits are not conclusive, as healthy rabbits exhibit remarkable variations in daily temperature, and finally that the hypodermic injection of human saliva will produce in the rabbit the same pathological effects as those which are alleged by Klebs and Crudeli to have followed from the introduction of the so-called malarial bacillus; in short, the investigations of this keen and crucial observer go far to show that the theories of Klebs and Crudeli are untenable, and that the clinical and pathological results they have alleged to have followed from the introduction of the bacillus are neither more nor less than the symptoms of blood poisoning.

Then the organism has not been found in other places where malarial fevers are prevalent.

Klebs and Crudeli affirm that the bacillus abounds in the blood of persons suffering from ague, but other observers have failed to discover it, and Dr. Oldham, of the Bengal Medical Service, has injected blood taken from a patient in this condition without effect into his own circulation. The verdict therefore which must be passed at present upon the theory of Klebs and Crudeli is that of the Scotch jury, which neither condemns nor acquits but leaves the matter not proven.

Consequently our attitude as to the determination of the malarial poison as a separate, particulate, and demonstrable material, must be one of expectancy, and an expectancy which has received an additional and very powerful stimulus by the investigations to which I have referred.

There are, however, some other characteristics of the malarial poison which appear to afford indirect evidence of its physical and material character, and one is especially the very strong proofs which have been adduced as to the poison being conveyed by the contamination of potable water. There are several interesting facts bearing upon this point; I will however confine myself to two instances recently published which appear to establish this fact in a remarkable manner.

One is a report on the prevalence of intermittent fever among the troops quartered at Tilbury Fort, on the left bank of the Thames nearly opposite Gravesend.

Tilbury Fort, in the Essex marshes, is supplied with *rain* water, which is collected on the roofs of various buildings and, before entering two large cemented tanks placed underground, passes through charcoal filters. A body of troops arrived at the fort on the 18th December, 1873, and remained until July, 1874. During this period the tanks were under repair and empty, and the troops were supplied with *spring* water for drinking and cooking purposes from the railway station. The tank water was not used at all. Only one case of ague occurred in the garrison in this period of seven months.

After the tanks had been repaired and came again into use, a fresh body of troops came to the garrison to relieve those already alluded to, and in the course of six months 12 per cent. of their number were attacked with intermittent fever. These troops used during this time *not* the spring water from the railway station, but the rain water from the underground tanks.

The fair assumption therefore was, that these rain-tanks being sunk in marshy ground, the malarial poison had percolated with the subsoil water through the walls of the tanks and contaminated the water, and that the malarial poison had thus gained access to the men. This assumption was strengthened by the fact that there were no other cases among the civil population in the neighbourhood at the time.

Another remarkable instance bearing upon this point has been recorded recently by Dr. Charles Smart, of the United States army.

In the Rocky Mountain regions of North America there prevails a fever which has received the popular name of "mountain" fever. The special outbreak to which I refer occurred at Fort Bridges, in Wyoming

Territory. Dr. Smart spent several months in the examination of the various springs, wells and river waters, used as potable waters by the civil and military population. The fever to which I allude has been spoken of as a malarial fever, a malarial remittent. Dr. Smart describes it as follows:—

1. A primary stage of one or two weeks, during which the individual is more or less oppressed by the influence of the *materies morbi*.

2. Development of fever, with irregular remission and much more depression and muscular prostration than the patient's pulse and temperature would lead the observer to expect.

3. A typhoid stage, marked by prostration, low delirium and coma-vigil.

This fever is very amenable to treatment by quinine. It is certainly not enteric fever. The symptoms and post-mortem appearances entirely exclude that. It could not be traced to local surroundings. The fact that it is rapidly cured by quinine gave strong assumption of its malarial origin, and yet there was nothing apparently malarious in the locality, which is 7,000 feet above the sea level. The question arose: how was the fever caused? After a long and patient investigation Dr. Smart came to these conclusions:—That the potable water used was taken from the mountain streams flowing from the snow ranges; that on analysis he found in this water a large amount of albuminoid ammonia; and that in the snow which forms the source of these streams he found a still larger amount, indicating the presence of a considerable quantity of organic matter. He concludes that the malarial poison is swept up from the plains with the organic matter, and is precipitated by the snow. He takes the organic matter as a measure of the malarious poison in water, just as carbonic acid is taken as measure of impurity in air, the intensity of the disease being a measure of the dose of the poison. The most remarkable fact is, that the cases coincide with the melting of the snow, and the locality is quite free from fever when there is no snow on the hills.

These interesting observations afford a tolerably clear assumption that the *materies morbi* of malarial fevers is an organic germ floating about in the air we breathe, and capable of introduction into the human system through the medium of the food we eat or the water we drink.

The pathological effects of the malarial poison upon the human organism are protean in their forms; varying from the intense saturation of the system, as evidenced by the most deadly form of paludal fever, attended with stupor, coma, and death in a few hours or days, to the gradual, slow, but sure process of infection exhibited among persons who have resided all their lives in marshy and malarious districts, and who present those signs of chronic malarial poisoning which once seen can never be forgotten.

Scarcely any organ in the body seems to escape the influence of this poison, and this too may be seen in persons who have never had an attack of ague or fever in their lives.

As an instance of the rapid and disastrous results of this poison in its most virulent form I may recall the celebrated Walcheren expedition in 1804. The expedition was one of the most magnificent which ever left the shores of England, and consisted of a force of 36,481 rank and file and 1,738 officers. The campaign, if such a name can be given to a military spectacle which lasted less than six months, and consisted in the occupation of a wretched little swampy island and the capture of one small town, was one of the most disastrous this country ever was engaged in from the effects of malarial poisoning. 12,000 fell sick in the island of Walcheren and 36,000 within six months after it was evacuated; within 50 days after the landing of the force on the banks of the Scheldt, there were from 7,000 to 10,000 men in hospital with malarial fever; the deaths were from 25 to 80 daily, and it was estimated that had the force remained in Walcheren for 250 days it would have been entirely destroyed. Those who escaped death in Walcheren, returned to England bringing with them the pathological evidences of the terrible nature of the poison to which they had been subjected; intermittent and remittent fevers, diarrhœa, dysentery, dropsy, liver and spleen enlargements.

This was an instance of malarial poisoning in its most virulent form.

I had an opportunity myself of observing the effects of slow malarial poisoning during a residence of four months in Bulgaria with the army under Lord Raglan. The troops were encamped on rough uncultivated ground, often in the vicinity of marshy lakes. There was great heat during the day with cold and dewy nights; fogs and mists prevailed, the food was insufficient and of inferior quality, and before long it was apparent that the strength of this splendid force was being gradually sapped. Many of the strongest became sickly without being able to define what was the matter with them; severe and fatal remittent fever prevailed. In the course of a few weeks regiments which, when they left England, seemed capable of going anywhere and doing anything, had become scarcely capable of accomplishing a march of five miles. The men were pale, weak and anœmic, and, in short, in the condition to which Vogel gives the term of malaria chlorosis. Men in such a condition fell an easy prey to the epidemic of Asiatic cholera which attacked them, and the force altogether became so enervated by chronic exposure to malarial poisoning, that in 22 days after landing in the Crimea 5,053 were disabled, of whom 353 were killed at the battle of the Alma, 1,867 were wounded, and 2,237 were sent sick to Scutari suffering from one or other of the many forms of malarial poisoning.*

No theory of chill, or sudden alteration of temperature, can account for such wide-spread attacks of illness among a body of men of such splendid physique, and so full apparently of life and vigour, as they were before they landed in Bulgaria.

This condition of malaria—chlorosis or malarial cachexia is observed principally among the inhabitants of notoriously malarial districts, such as the Maremma of Italy, the lower Alps, the great Sunderbund near Calcutta. "A glance at such inhabitants," says Dr. James Johnson, "shows that the range of disorders produced by the poison of malaria is very extensive. The jaundiced complexion, the stunted growth, the stupid countenance, the distended abdomen, and often the dropsical limbs, and shortened life, attest that habitual exposure to this poison saps the energy of every bodily and mental function, and drags its victim to an early grave.

"Fever and ague are as a drop of water in the ocean compared with the other less prominent but more dangerous maladies that silently but effectually disorganise the vital structures under the operation of this deleterious and invisible poison."

Shakespeare seems to have had before his mind's eye

* I am indebted for these facts to my friend Professor Aitken, Army Medical School, Netley.

such a victim, when in the play of the *Tempest* he puts into the mouth of Caliban such an horrible invocation as this:—

> "All the infections that the sun sucks up
> From bogs, fens, flats, on Prosper fall, and make him
> By inch-meal a disease!"

Let us glance for a moment at the effect of the malarial poison upon the blood. It is here perhaps that its influence is felt first, and when this influence is prolonged, its action as a destroyer of red corpuscles is clearly discernible, not only clinically but by actual enumeration.

Cornil and Ranvier thus describe the effects:—"In simple intermittent fever there is after each access a considerable diminution of the number of red corpuscles. Repair then takes place, but it has not time to be accomplished before the return of the next access, and thus the anæmia becomes progressive. As recovery is approached the reverse is the case. The attacks of fever becoming less frequent, time is given for the reconstruction of corpuscles, the blood recovers more than it has lost."

The white corpuscles also diminish in numbers after each access of fever more in proportion than do the red, this is especially so in the condition of malarial cachexia to which I have adverted.

In reference to the question of melanæmia and pigmentation generally it would seem from recent observation that this peculiar alteration or degeneration takes place primarily in the spleen; at all events it is observed most frequently in this organ after it has undergone a series of congestive attacks. The particles of pigment met with in the blood under these conditions are described as being round or angular and of a very black colour; they appear to be taken up by the white corpuscles, and are carried to the different organs.

I may here allude to the experiments of Cuboni and Marchiafava on the blood of malarial patients. They found frequently if not constantly, and in considerable numbers, minute bodies—micro-organisms—of a spherical shape, and invariably placed in the centre of the white corpuscles. At the beginning of an attack of fever bacilli were seen bearing a spore at each end, their length from one to three times the diameter of a red corpuscle. The bacilli increased during an attack of fever, and the spores became more abundant. These observations have been confirmed by Lanze and Peroncilo.

The most recent observations have been reported by Laveran, in 1880, in Algeria and Tunis. He examined the blood of 192 patients suffering from various forms of malarial fever, and in 180 he found cylindrical curved bodies, also spheroids, the size of a corpuscle, containing pigment grains. Besides this the blood contained pigmented red corpuscles and free pigment granules. The addition of a minute quantity of a solution of quinine destroyed these organisms.

The observations of Richard, of Philipsville, seems to point to the presence of a parasite in the blood of malarial patients. He has observed its life history; he considers the red corpuscle to be its special habitat, in which it developes and leaves at maturity. During the paroxysms of fever the corpuscle presents, according to this observer, a round spot, which rapidly enlarges and is encircled by round black granules. Ultimately the hæmoglobin round this substance disappears, leaving a body the size of a corpuscle, containing a collarette of black granulations.

Upon these observations Richard founds the hypothesis that the formidable cerebral symptoms observed in acute malarial poisoning—the vomiting, violent delirium and subsequent coma, may be produced by the blocking of the cerebral capillaries by these granular pigmentary bodies. If these observations be true, they may account for other clinical symptoms of malarial poisoning, the ancemia arising from enormous destruction of red corpuscles; the persistence of the infection remaining dormant in the system sometimes for a period of twenty years, and breaking out afresh under special exciting causes, a persistence which seems to be approached only by that of the syphilitic virus.

Now it must be confessed that although these observations on the condition of the blood in malarial poisoning have been conducted with great care, there is a certain vagueness and obscurity about them, and their hyper-scientific character renders any satisfactory conclusions difficult and conflicting. But I think at all events we may draw from them this inference; that whatever the physical characters of the malarial poison may be, whether it is a bacillus as Crudeli avers, or a germ so minute as to defy the powers of the microscope—there can be very little doubt that its action as a blood disintegrator and destroyer is based upon tolerably clear evidence.

If we believe malarial fevers to result from a special blood infection, the secondary effects of the poison upon the different systems and organs of the body are not difficult to account for. These effects are probably most manifest in the organs engaged especially in the formation of the blood, notably the spleen, liver and lymphatic system, as well as those engaged in the processes of excretion, such as the kidneys, intestinal tract and skin.

The spleen suffers more or less in all fevers. Perhaps we do not pay so much attention to this fact as do our brethren of the German school. Professor Friedrich, of Heidelberg, maintains that this organ undergoes enlargement in all infectious fevers, in all those concerning the development of which there is fair ground for assuming the existence of a contagium vivum of some sort. At all events in the vast majority of malarial fevers the spleen undergoes important pathological changes, probably the result of concentration within its vascular pulp of poisonous material, which by its irritating properties produces enormous hyperplastic proliferation of this element of the splenic structure.

The malarial poison has, if I may use the expression, a special affinity for the spleen. When the poisonous dose is large and suddenly imbibed, the changes produced in the spleen are extremely rapid. Instances have occurred where persons have died in a few hours from the intensity of the dose of malaria, and in such cases the spleen, liver and intestinal tract have all been found intensely congested, but especially the spleen.

We are all familiar with the effect of chronic malarial poisoning upon the spleen, the chronic enlargement and hardening, and the formation of the well-known ague-cake, with all its disastrous consequences of hæmatemesis, dropsy and lingering death.

The size which the spleen attains in these latter cases is sometimes enormous. In the Pathological Museum of the Army Medical Department there is a spleen taken from the body of a drummer boy, aged 14, who had been only two years in India. When he returned to England his abdomen was so large and tense that he was supposed to be suffering from ascites; he was tapped without any result. He died subsequently, and at the autopsy the spleen was found extending from the false ribs to the ilium; it weighed 10 lbs. 15 oz.; it measured 14 in. by $7\frac{3}{4}$ in., and it was nearly 5 in. in thickness.

Friedrich states that splenic enlargement may be accounted for on the hypothesis of a substantia viva; that the contagium being carried into the vascular apparatus of the organ accumulates there, and attains a degree of intensity and concentration varying according to the dose of the poison, producing alteration of the pulp and general hypertrophy.

Hertz attempts to explain the action of the poison more minutely; he thinks its influence on the spleen may be accounted for on the supposition that on the one hand it paralyses the motor nerves of the coats of the vessels, thus inducing hyperæmia; on the other hand that it directly injures the blood in the vessels and in the parenchyma of the gland. By the latter means the red corpuscles are disintegrated and transformed into pigment, and the white corpuscles are hindered in their development.

If it be true that the spleen has this faculty of retaining and holding within it the ultimate factors of acute febrile disease, may it not be reasonably entertained that the malarial contagium finds in this organ a special nidus which supports its vitality and stores up its power, keeping it dormant for a time, and again suffering it to burst into life and activity under circumstances favourable to its development; thus accounting for the periodical storms of fever at long intervals occurring in persons who had at some previous part of their lives been subject to the influence of the poison.

Although it may be said that splenic congestion is the special lesion of malarial poisoning, other organs suffer in a greater or lesser degree, specially the liver and intestinal tract. But it has a much more extensive influence than this. Dr. Norman Chevers considers that many of the gravest diseases of India are largely due to malaria. Cholera, dysentery, suppurative hepatitis, land scurvy, sloughing of the cornea, menorrhagia, a tendency to abort, post partum hæmorrhage, tetanus, rheumatism, bowel complaints, are all, he says, chargeable to the account of malarial poisoning.

Dr. Maclagan in his recent work on Rheumatism discusses his reasons for believing that the malarial poison is largely concerned in producing the disease, and that the poison is an organism whose morbific action is intimately associated with its organic development. Whether the miasmatic theory of rheumatic fever be the true one or not, there is at all events a certain analogy between the features of rheumatic and those of malarial fevers to which Dr. Maclagan draws attention, as for example: The non-elimination of the poison; its persistence and lengthened vitality within the body; the non-contagious character of the fever and the liability of the patient to repeated attacks.

And bearing upon this point I may add that some of the most intractable forms of rheumatism, both articular and muscular, with which I have ever met, have been preceded by malarial intermittent or remittent fever, notably those occurring in Malta and Gibraltar.

Time will not permit me to discuss the sequelæ of malarial poisoning, its effects upon the kidneys and genito-urinary passages, evidenced by such phenomena as intermittent albuminuria and hæmaturia, or its baneful results on the brain and nervous system, and the numerous forms of neuralgia which spring from its influence.

I suppose I am right in the assumption that one of the most cogent proofs of the existence of a specific poison-germ or contagium vivum, or by whatsoever name you may choose to call it, lies in the clinical fact of a large number of persons being struck down within a given area by a fever presenting similar symptoms in each case. Take for example such an instance as that which occurred at Umritsir, a large and populous city in the Punjab, in 1881. Scarcely an individual in the city escaped an attack, business was almost suspended, nine-tenths of the shops were closed. The death-rate rose to 200 a day. The type of the fever presented all the recognised malarial characters; intermittent and remittent, attended with bilious vomiting and splenic enlargement. The mortality among children was excessively high; out of a total mortality of 6,859 there were 3,531 deaths among children, showing the marked susceptibility of children to the malarial poison.

The health officer of the station attributed the outbreak to a heavy rain-fall, water-logging of the soil and obstructed sewerage; most probably also where the fever was so universal as in this case the drinking water became contaminated with the malarial poison-germ. There seems to have been no idea entertained that the fever was in any way contagious. In its clinical aspects and pathological lesions it was a malarious fever pure and simple.

Finally, have we not another argument in favour of the germ theory of malarial fevers in the fact of their being generally amenable to treatment by quinine? How does quinine act? We know that it is an anti-pyretic and anti-periodic. We give it in large doses in fevers and other diseases attended with hyper-pyrexia; and we do so with confidence. We know almost to a certainty that it will answer our expectations. For if there be such a thing as a specific remedy, it is surely quinine in the treatment of malarial fevers. But by what power it acts in this way we know not. In the absence of proof to the contrary it is at least justifiable to surmise that it acts by its antiseptic properties, by its destructive agency on the specific germs of fever in the blood.

If we accept the theory of a special malarial poison, it is not unreasonable to assume that quinine exercises its special curative power in rendering this poison inoperative within the body, and its special prophylactic power by rendering the blood unfit for its reception and development.

Briefly then to sum up the proofs of the existence of a special malarial poison germ, short of its actual demonstration, we may thus express them :—

First.—Malarial fevers have a period of incubation followed by fever-storm.

Second.—They have special organic lesions.

Third.—The destructive effects of the poison upon the blood are demonstrable by actual observation.

Fourth.—The evidence of the communicability of the poison through potable water is, to say the least, very strong.

Last.—The influence of antidotes, such as quinine, arsenic, eucalyptus, in counteracting the effects of the poison is clearly manifest.

I cannot however bring the subject to a close without alluding to the efforts which are being made, at least in one notorious locality, to meet and fight the enemy on its own ground.

The Roman Campagna, in long ages past the site of large cities, has been notorious for 2,000 years for its malarious fevers. Who does not think twice even now about advising a friend or a patient to pass a winter in Rome? The ancient inhabitants of Rome knew the dangers which were close to their walls; a whole system of drainage has been recently discovered in this locality, which has arisen as it were from the grave of a long buried past to rebuke with bitter irony the apathy of the present generation. It seems, however, that at last something in the way of practical effort is about to be made to mitigate the dangers of this notorious plague spot. The late General Garibaldi, unpractical and visionary as he may have been, fervently desired the removal of the evil, and conceived and agitated some wild scheme for the drainage of the Campagna.

The Trappist monks, whose mission in life is prayer and the tilling of the soil, have been at work for some years in the locality. The Italian Government gave them a large tract of ground on condition that they planted 100,000 trees in ten years. They established the farm of the Tre Fontane and began planting the eucalyptus; thousands of these trees are growing now, and their effect upon the subsoil water is remarkable. In addition to this there is a Government scheme, proposed it is said at the instance of Crudeli, for the gradual drainage of the Campagna, which, if carried out, will doubtless produce admirable sanitary results, although the undertaking is a gigantic one.

The prevalence of malarial fevers in England is a matter of past history; an advanced civilization, extensive drainage, a purer water supply, have combined to sweep away a form of fever which in the days of Sydenham was a veritable plague. It lingers still in some spots, notably in the Isle of Sheppey, and there are few practitioners who do not meet with occasional cases.

And now, gentlemen, having thus very imperfectly discussed before you the special subject of this address, allow me in conclusion to make a few general remarks.

The report of our Society, which will presently be read, will tell you of our increasing numbers, of the interest and value of the papers which have been read, and the morbid specimens which have from time to time been demonstrated for our instruction.

I can truly say for myself that I never attended a meeting of this Society without learning something I did not know before. The advances of surgical and medical science are a marvel to the world. When I reflect upon the surgery and the practice of medicine of nearly forty years ago, and see what is done to-day, I feel like one who has awaked from a dream.

I can recall the time when surgeons shrank with a feeling akin to terror from the thought of wounding the peritoneum, and I have lived to see the wonders now accomplished in the special field of abdominal surgery, and in other branches of the art.

I was invited some months ago by the kindness of one of our members to witness an ovariotomy. The ovarian tumour was found to be firmly and extensively adherent to the uterus, from which it was separated with very great difficulty; the uterus itself, torn, and bleeding profusely from many points, was turned out on the abdominal wall, a large number of vessels on its surface were ligatured, and the womb in this condition was returned to its place —and the patient recovered.

I was permitted by the kindness of another friend to witness an operation performed by him for the cure of deformed legs, and I saw him deliberately manufacture five compound factures while the patient was on the operating table: and no harmful, but good, results followed. To you who are hospital surgeons, and are seeing and doing such things daily, these may not seem extraordinary facts; I, who have ceased to practise surgery for many years, regard them as among the marvels of the time. When I recall the unsatisfactory results which followed the treatment of empyema in former years, and contrast them with those which are obtained from a more rational method by free incision of the pleural cavity under antiseptic precautions, and an improved method of drainage; and beside all this, when I observe the advanced and still advancing steps in practical medicine, especially in the direction of greater accuracy in diagnosis by physical means, by the aid of the thermometer, the ophthalmoscope, the laryngoscope, and the sphygmograph, I am filled with wonder and thankfulness that our noble profession has made such strides. And how have these things been accomplished? By concentrated effort individually and collectively, by mutual help and encouragement, by the intellectual attrition among the members of our profession as a body, which clears away the useless and the effete, and leaves only the pure gold of fact and truth.

The value of societies like ours has been proved over and over again, but I think we may claim for our own that it is purely scientific. I have never heard a subject of medical ethics discussed here; better avoid them. We come here to learn and to teach, and if the oldest and wisest amongst us can unfold treasures from the rich store of their matured experience, there is not one, however experienced he may be, who may not learn something from the lips of our very youngest member. Our Society has passed almost through its first Decade; that period has not been unfruitful, and we start afresh now with a new interest, with a young and, I venture to think, a vigorous child to support in the Journal which we have undertaken to foster. We have provided our fledgling with the healthiest surroundings, in the shape of a good committee and a most able and indefatigable editor; but it is for you, gentlemen, and, we trust, for our brethren in the West, to supply it with food suitable for its nourishment, so that it may grow into a strong and influential manhood.

We meet here for practical work and study, in order that we may gather from each other's experience that knowledge which will enable us the better to fight the battle which we wage, however feebly, against disease and death; to learn more of the ever-varying morbid processes constantly operating upon the different organs and structures of the human body, and to look as deeply as we can into those mysteries of undiscovered truth, to attempt the solution of which has been the endeavour of our art from remote ages to the present hour.

Not in the world of light alone,
Where God has built His blazing throne,
Nor yet alone on earth below,
With belted seas that come and go,
And endless isles of sunlit green,
Is all thy Maker's glory seen;
Look in upon thy wondrous frame—
Eternal wisdom is the same.

